# Clinical outcomes in non-small cell lung cancer patients with an ultra-high expression of programmed death ligand-1 treated using pembrolizumab as a first-line therapy: A retrospective multicenter cohort study in Japan

**DOI:** 10.1371/journal.pone.0220570

**Published:** 2019-07-31

**Authors:** Ryuya Edahiro, Masaki Kanazu, Hiroyuki Kurebe, Masahide Mori, Daichi Fujimoto, Yoshihiko Taniguchi, Hidekazu Suzuki, Katsuya Hirano, Toshihide Yokoyama, Mitsunori Morita, Yasushi Fukuda, Junji Uchida, Takeshi Makio, Motohiro Tamiya

**Affiliations:** 1 Department of Thoracic Oncology, National Hospital Organization Osaka Toneyama Medical Center, Toyonaka, Japan; 2 Department of Respiratory Medicine, Kobe City Medical Center General Hospital, Kobe, Japan; 3 Department of Internal Medicine, National Hospital Organization Kinki-Chuo Chest Medical Center, Osaka, Japan; 4 Department of Thoracic Oncology, Osaka Habikino Medical Center, Osaka, Japan; 5 Department of Respiratory Medicine, Hyogo Prefectural Amagasaki General Medical Center, Amagasaki, Japan; 6 Department of Respiratory Medicine, Kurashiki Central Hospital, Kurashiki, Japan; 7 Department of Respiratory Medicine, Kobe City Medical Center West Hospital, Kobe, Japan; 8 Department of Respiratory Medicine, Himeji Medical Center, Himeji, Japan; 9 Department of Respiratory Medicine, Osaka General Medical Center, Osaka, Japan; 10 Department of Respiratory Medicine, Itami City Hospital, Itami, Japan; 11 Department of Thoracic Oncology, Osaka International Cancer Institute, Osaka, Japan; College of Medicine, National Cheng Kung University, TAIWAN

## Abstract

**Background:**

Pembrolizumab is currently approved as a first-line therapy for advanced non-small cell lung cancer (NSCLC) patients with a programed death ligand-1 (PD-L1) expression ≥50%. However, the association between the efficacy of pembrolizumab and PD-L1 expression levels in patients with PD-L1 expression ≥50% has not been fully elucidated.

**Methods:**

We retrospectively analyzed patients with advanced NSCLC and a PD-L1 tumor proportion score (TPS) of ≥50% who received pembrolizumab as a first-line therapy at 11 institutions in Japan between February 2017 and January 2018. Patients were divided into TPS 50–89% and TPS 90–100% (ultra-high PD-L1 expression) cohorts.

**Results:**

In total, 149 patients were included: 99 (66.4%) and 50 (33.6%) patients were in the TPS 50–89% and TPS 90–100% cohorts, respectively. Baseline characteristics were similar between the TPS 90–100% and TPS 50–89% cohorts. The objective response rates (ORR) in the TPS 90–100% and TPS 50–89% cohorts were 58.0% and 46.5%, respectively (*p* = 0.23). Time to treatment failure (TTF) was longer in the TPS 90–100% cohort than in the TPS 50–89% cohort (hazard ratio [HR]: 0.67, 95% confidence interval (CI): 0.42–1.07; *p* = 0.09). Although TTF within 120 days after the initiation of pembrolizumab therapy was comparable between both cohorts (*p* = 0.54), TTF after 120 days was significantly longer in the TPS 90–100% cohort than in the TPS 50–89% cohort (HR: 0.22, 95% CI: 0.06–0.87; *p* = 0.031). Immune related adverse events of grade 3 or more occurred in 16.0% and 19.2% of patients in the TPS 90–100% and TPS 50–89% cohorts, respectively.

**Conclusions:**

The patients with an ultra-high PD-L1 expression continued pembrolizumab therapy longer, driven by a reduced risk of treatment failure in the late phase. PD-L1 expression levels might be a predictive biomarker of a first-line immunotherapy benefit in the late phase among NSCLC patients with TPS ≥50%.

## Introduction

Lung cancer is the leading cause of cancer-related death worldwide [1]. Non-small cell lung cancer (NSCLC) accounts for approximately 80% of all lung cancer cases and the majority of these cases are diagnosed at an advanced stage [2]. Some molecular targeting agents such as epidermal growth factor receptor (EGFR) tyrosine kinase inhibitors (TKIs) and anaplastic lymphoma kinase (ALK) inhibitors, which have been found to provide high response rate in patients with EGFR or ALK mutations [3,4], dramatically changed the treatment strategy for advanced NSCLC. However, most patients with NSCLC do not harbor these oncogenic drivers, and treatment options have been limited to cytotoxic chemotherapy for these patients.

Recently, immune-checkpoint inhibitors (ICIs) have been established for several cancers, such as advanced melanoma and NSCLC [5–7]. Particularly, the efficacy of pembrolizumab for first-line treatment of NSCLC patients with a programmed death ligand 1 (PD-L1) tumor proportion score (TPS) more than 50% was reported [7,8]. Additionally, pembrolizumab is widely used for this population. However, the clinical benefit of pembrolizumab was only found for less than half of the patients with a PD-L1 TPS more than 50% [8,9]. Therefore, there is an urgent need to develop clinically practical tools to identify the subgroup of patients most likely to derive clinical benefits from ICIs [8–10].

Although a higher PD-L1 expression is demonstrated to correlate with a higher response rate in patients treated with pembrolizumab [7,8,10,11], the association between clinical outcomes of ICIs and PD-L1 expression levels in NSCLC patients with a high PD-L1 expression has not been extensively investigated. Therefore, we conducted a retrospective multicenter cohort study to evaluate the association between the efficacy and tolerability of pembrolizumab and PD-L1 expression levels among patients with a PD-L1 TPS ≥50%.

## Materials and methods

### Patients and treatment

This retrospective multicenter cohort study included advanced NSCLC patients (unresectable stage III or IV, and recurrence after operation or radiotherapy, based on the 7^th^ edition of the TNM classification) with a PD-L1 TPS ≥50% who received first-line pembrolizumab at any of the 11 participating institutions in Japan between February 2017 and January 2018. Patients were excluded if they had an autoimmune disease requiring systemic treatment. However, patients with type 1 diabetes mellitus or hypothyroidism, which were manageable by hormone replacement therapy and those with cutaneous disease, which did not require systemic treatment were included. Patients were eligible for inclusion in the present study if their PD-L1 TPS was categorized into 50–89% or 90–100%. PD-L1 expression was assessed in formalin-fixed tumor samples with the use of the commercially available PD-L1 IHC 22C3 pharmDx assay (Dako North America) at each institution. Informed consent was obtained in the form of opt-out on a dedicated website. The data were analyzed anonymously. The study protocol was approved by the review board of each institution (National Hospital Organization Osaka Toneyama Medical Center, Kobe City Medical Center General Hospital, National Hospital Organization Kinki-Chuo Chest Medical Center, Osaka Habikino Medical Center, Hyogo Prefectural Amagasaki General Medical Center, Kurashiki Central Hospital, Kobe City Medical Center West Hospital, Himeji Medical Center, Osaka General Medical Center, Itami City Hospital and Osaka International Cancer Institute) and is registered with UMIN (University Hospital Medical Information Network in Japan; number 000032470).

### Outcome measures

In all patients, the Eastern Cooperative Oncology Group (ECOG) performance status (PS) was evaluated just prior to commencing pembrolizumab. Treatment response was assessed according to the Response Evaluation Criteria in Solid Tumors (RECIST version 1.1) [12]. The interval between the date of commencing pembrolizumab treatment and that of treatment failure (TTF) and disease progression/death (progression-free survival; PFS) was calculated for each patient.

Immune related adverse events (irAEs) were defined as adverse events (AEs) with a potential immune-mediated etiology that may require immune-modulating or endocrine therapy. Early irAEs were defined as irAEs that occurred within 3 weeks after commencing pembrolizumab treatment. AEs were graded according to the National Cancer Institute Common Terminology Criteria for Adverse Events, version 4.0.

### Statistical analysis

Continuous and categorical data were summarized as medians (range) and numbers (percentages) and were compared between patients with PD-L1 TPS 90–100% and those with PD-L1 TPS 50–89% using Wilcoxon rank-sum and Fisher's exact tests, respectively. The Kaplan-Meier method was used to estimate survival curves of TTF and PFS according to PD-L1 expression levels (TPS 90–100% versus [vs] TPS 50–89%). A univariate Cox proportional hazards regression model was used to estimate hazard ratios (HRs) and 95% confidence intervals (CIs). The rates of treatment failure free survival and progression free survival were estimated at 120 and 365 days, respectively, and landmark analyses were performed after 120 days of follow-up to assess the risk of treatment failure and disease progression at the late phase in the TPS 90–100% and TPS 50–89% cohorts.

All reported p-values were two sided, and values of *p*<0.05 were considered statistically significant. SAS software version 9.3 (SAS Institute, Inc., Cary, NC) was used for statistical analysis.

## Results

### Patients’ characteristics

The baseline characteristics for all patients and comparisons between the patients in the PD-L1 TPS 90–100% and the PD-L1 TPS 50–89% cohorts are presented in Table 1. Overall 149 patients were included in the analysis; the median age was 71 years, 124 (83.2%) patients were men, and 118 (79.2%) patients had an ECOG PS of 0–1. Ninety-nine (66.4%) and 50 (33.6%) patients were in the TPS 50–89% and TPS 90–100% cohorts, respectively. There were no significant differences in the baseline clinical characteristics between the TPS 90–100% and TPS 50–89% cohorts.

**Table 1:** The baseline characteristics in patients based on PD-L1 expression level.

		TPS 90–100% cohort	TPS 50–89% cohort	Total	
Characteristics		(N = 50)	(N = 99)	(N = 149)	*p* value
Age—yr					
	Median (range)	71 (47–82)	71 (39–87)	71 (39–87)	0.72
Male—no. (%)		40 (80.0)	84 (84.9)	124 (83.2)	0.49
Smoking status—no. (%)					
	Never	4 (8.0)	11 (11.1)	15 (10.1)	0.77
	Current or former	46 (92.0)	88 (88.9)	134 (89.9)	
ECOG PS—no. (%)					
	0	13 (26.0)	19 (19.2)	32 (21.5)	0.55
	1	26 (52.0)	60 (60.6)	86 (57.7)	
	2	8 (16.0)	16 (16.2)	24 (16.1)	
	≧3	3 (6.0)	4 (4.0)	7 (4.7)	
Histological type—no. (%)					
	Squamous	9 (18.0)	26 (26.3)	35 (23.5)	0.31
	Non-Squamous	41 (82.0)	73 (73.7)	114 (76.5)	
EGFR mutation—no. (%)					
	Positive	2 (4.0)	4 (4.0)	6 (4.0)	1.00
	Negative	42 (84.0)	83 (83.8)	125 (83.9)	
	Not evaluated	6 (12.0)	12 (12.1)	18 (12.1)	
EML4-ALK translocation—no. (%)					
	Positive	0 (0.0)	0 (0.0)	0 (0.0)	1.00
	Negative	43 (86.0)	85 (85.9)	128 (85.9)	
	Not evaluated	7 (14.0)	14 (14.1)	21 (14.1)	
Stage—no. (%)					
	III	9 (18.0)	21 (21.2)	30 (20.1)	0.55
	IV	37 (74.0)	65 (65.7)	102 (68.5)	
	Recurrence	4 (8.0)	13 (13.1)	17 (11.4)	

Categorical variables are presented as number (percentage), and continuous variables are presented as median and range. Categorical variables were compared by Fisher's exact test, and continuous variables were compared by the Wilcoxon rank sum test for two-group comparisons. PD-L1 = programmed death ligand-1, TPS = tumor proportion score, ECOG PS = Eastern Cooperative Oncology Group performance status, EGFR = epidermal growth factor receptor, EML4-ALK = echinoderm microtubule-associated protein-like 4 (EML4) and anaplastic lymphoma kinase.

### Clinical outcomes according to PD-L1 tumor proportion score level

The best response, objective response rate (ORR), and disease control rate (DCR) for all patients and based on PD-L1 TPS grouping are summarized in Table 2. Among all patients, the ORR and DCR were 50.3% and 72.5%, respectively. The ORR and DCR did not significantly differ between patients in the TPS 90–100% and TPS 50–89% cohorts (58.0% vs 46.5%, *p* = 0.23; 76.0% vs 70.7%, *p* = 0.56, respectively).

**Table 2:** Clinical outcomes of pembrolizumab monotherapy according to PD-L1 expression level.

		TPS 90–100% cohort	TPS 50–89% cohort	Total	
Response		(N = 50)	(N = 99)	(N = 149)	*p* value
Best response—no. (%)					
	Complete response	1 (2.0)	1 (1.0)	2 (1.3)	0.49
	Partial response	28 (56.0)	45 (45.5)	73 (49.0)	
	Stable disease	9 (18.0)	24 (24.2)	33 (22.2)	
	Progression disease	12 (24.0)	25 (25.3)	37 (24.8)	
	Not evaluated	0 (0.0)	4 (4.0)	4 (2.7)	
Objective response rate—no. (%)		29 (58.0)	46 (46.5)	75 (50.3)	0.23
Disease control rate—no. (%)		38 (76.0)	70 (70.7)	108 (72.5)	0.56

Complete and partial responses were assessed by the investigator according to the Response Evaluation Criteria in Solid Tumors, version 1.1. *p* values were calculated using Fisher's exact test. PD-L1 = programmed death ligand-1, TPS = tumor proportion score.

**Fig 1:**
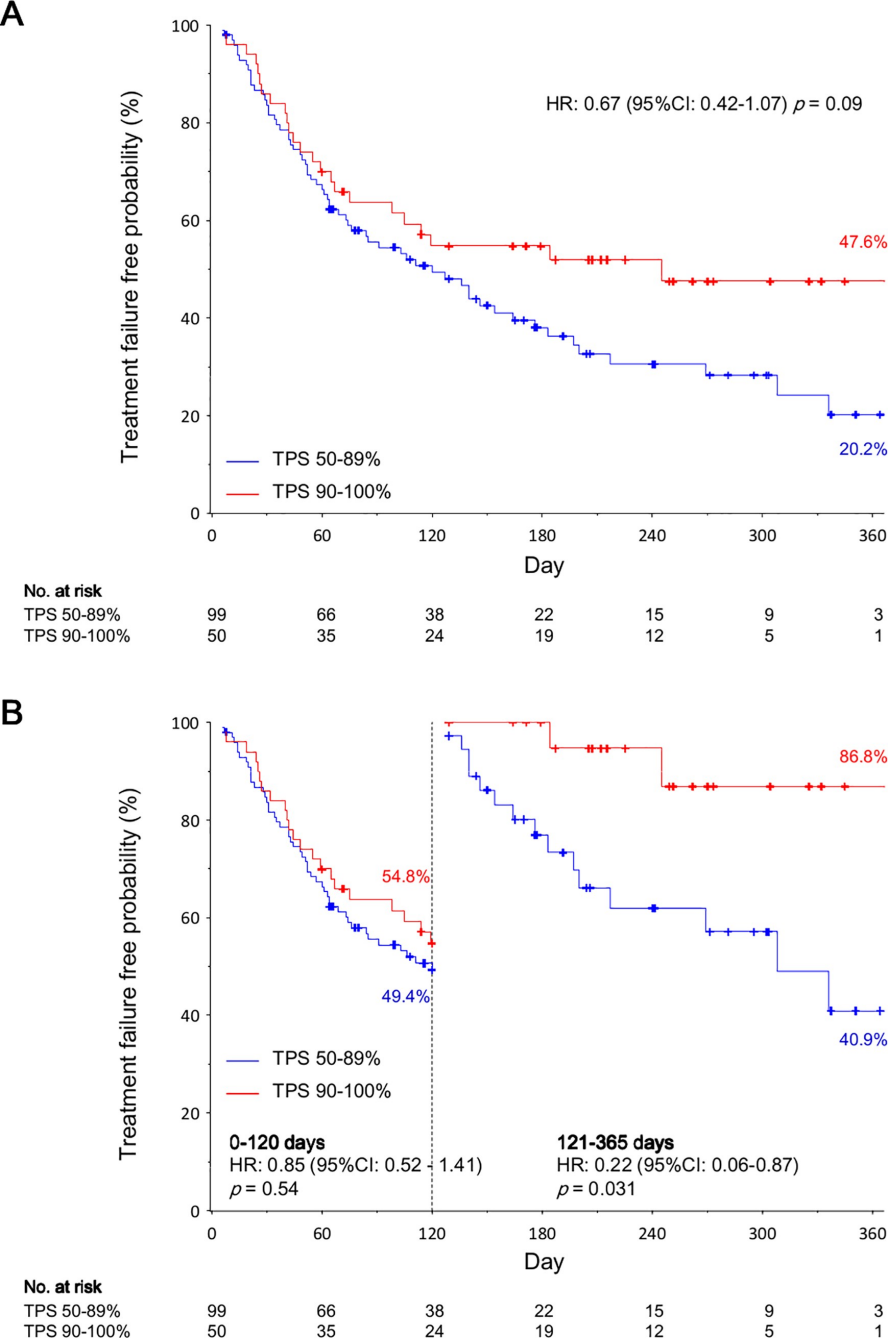
Time to treatment failure of pembrolizumab: the tumor proportion score 90–100% cohort versus the tumor proportion score 50–89% cohort. Panel A shows the Kaplan–Meier survival curves for the time to treatment failure according to the programed death ligand-1 expression levels for the tumor proportion score 90–100% cohort versus the tumor proportion score 50–89% cohort. Panel B shows the Kaplan–Meier survival curves for time to treatment failure before and after 120 days according to the programed death ligand-1 expression levels in landmark analyses. Hazard ratios are for the tumor proportion score 90–100% cohort versus the tumor proportion score 50–89% cohort. The hazard ratios, 95% confidence intervals, and p-values were calculated using univariate Cox regression analysis. Cross marks represent data censored at the last time the patient was known to be alive. Abbreviations: HR, hazard ratio; TPS, tumor proportion score.

The treatment failure free survival rate at 365-days of follow-up was 47.6% and 20.2% in the TPS 90–100% and the TPS 50–89% cohorts, respectively. TTF was longer in the TPS 90–100% cohort than in the TPS 50–89% cohort (HR: 0.67, 95% CI: 0.42–1.07; *p* = 0.09) (Fig 1A). TTF within 120 days after commencing pembrolizumab therapy was comparable between both cohorts (HR: 0.85, 95% CI: 0.52–1.41; *p* = 0.54). However, TTF after 120 days was significantly longer in the TPS 90–100% cohort than in the TPS 50–89% cohort (HR: 0.22, 95% CI: 0.06–0.87; *p* = 0.031) (Fig 1B).

PFS was not significantly longer in the TPS 90–100% cohort than in the TPS 50–89% cohort (HR: 0.78, 95% CI: 0.48–1.29; *p* = 0.34) (S1A Fig). However, PFS after 120 days was longer in the TPS 90–100% cohort than in the TPS 50–89% cohort (HR: 0.39, 95% CI: 0.14–1.13; *p* = 0.08) (S1B Fig).

### Subgroup analysis of HRs for TTF: PD-L1 tumor proportion score 90–100% vs 50–89%

**Fig 2:**
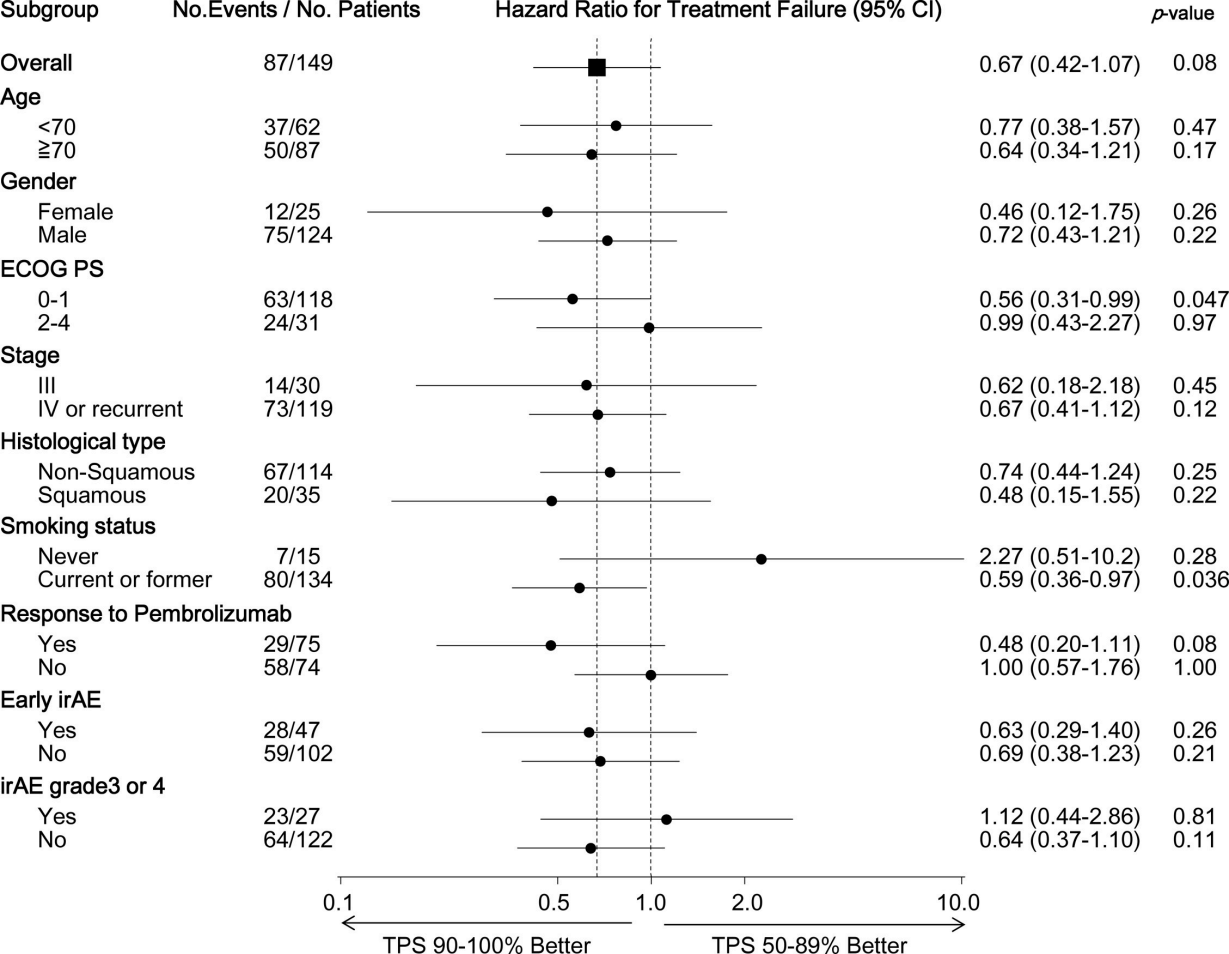
Exploratory subgroup analyses of time to treatment failure: The tumor proportion score 90–100% cohort versus the tumor proportion score 50–89% cohort. Hazard ratios for the time to treatment failure in the tumor proportion score 90–100% cohort versus the tumor proportion score 50–89% cohort. The hazard ratios, 95% confidence intervals, and p-values for each key subgroup were calculated using univariate Cox regression analysis. The response to pembrolizumab was defined as complete and partial responses assessed by the investigator according to the Response Evaluation Criteria in Solid Tumors, version 1.1. Early irAE was defined as irAE that occurred within 3 weeks after commencing pembrolizumab therapy. Abbreviations: HR, hazard ratio; TPS, tumor proportion score; ECOG PS, Eastern Cooperative Oncology Group performance status; irAE, immune-related adverse event.

HRs for the TTF of the TPS 90–100% cohort vs the TPS 50–89% cohort in various subgroups are presented in Fig 2. The benefit of pembrolizumab in the TPS 90–100% cohort over the TPS 50–89% cohort was observed in all subgroups, with the exception of patients who never smoked and those who had an irAE of Grade 3 or more (HR = 2.27, 95% CI: 0.51–10.2; *p* = 0.28, and HR = 1.12, 95% CI: 0.44–2.86; *p* = 0.81, respectively). Among the patients who experienced a response to pembrolizumab, the patients in the TPS 90–100% cohort derived a longer clinical benefit from pembrolizumab than those in the TPS 50–89% cohort (HR: 0.48, 95% CI: 0.20–1.11; *p* = 0.08). However, this was not evident among those who did not experience a response to pembrolizumab (HR: 1.00, 95% CI: 0.57–1.76; *p* = 1.00, S2 Fig).

### Frequency of irAE according to PD-L1 tumor proportion score

Table 3 shows the frequency of irAEs in the TPS 90–100% and TPS 50–89% cohorts. The proportion of early irAEs in the TPS 90–100% and the TPS 50–89% cohorts were 36.0% and 29.3% (*p* = 0.46), and that of irAEs of Grade 3 or more according to CTCAE ver. 4.0 were 16.0% and 19.2% (*p* = 0.82), respectively. Discontinuation rates of pembrolizumab due to irAEs were slightly lower in the TPS 90–100% cohort than the TPS 50–89% cohort (8.0% vs 19.2%, *p* = 0.09).

**Table 3:** Immune-related adverse events after commencing pembrolizumab therapy.

	TPS 90–100% cohort	TPS 50–89% cohort	
Adverse event	(N = 50)	(N = 99)	*p* value
Early irAE—no. (%)	18 (36.0)	29 (29.3)	0.46
irAE grade 3 or 4—no. (%)	8 (16.0)	19 (19.2)	0.82
irAE leading to withdrawal from treatment- no. (%)	4 (8.0)	19 (19.2)	0.09

irAE = immune-related adverse event. Early irAE was defined as irAE that occurred within 3 weeks after commencing pembrolizumab therapy. *p* values were calculated using Fisher's exact test. TPS = tumor proportion score.

## Discussions

This retrospective study demonstrated that patients with an ultra-high expression of PD-L1 were able to continue first-line pembrolizumab therapy longer for advanced NSCLC. The treatment failure-free benefit for patients with a TPS 90–100% emerged in the late phase, 120 days after commencing pembrolizumab therapy. We also revealed that pembrolizumab had a higher response rate among patients with a TPS 90–100% when compared to those with a TPS 50–89% (58.0% vs 46.5%). These data indicate that higher PD-L1 expression levels could be a predictive biomarker for the benefit of pembrolizumab therapy among NSCLC patients with TPS ≥50%.

The treatment failure-free survival rate at 365 days among the TPS 90–100% cohort was double that of the TPS 50–89% cohort (47.6% vs 20.2%). This was driven by a reduced risk of treatment failure in the late phase (after 120 days), because TTF within the first 120 days was comparable between both cohorts. This trend was also observed in PFS analysis. HR of TTF in the late phase was the smallest when the cut-off value was 120 days compared with when the cut-off value was 60 days or 180 days (S3 Fig). The late phase benefit of pembrolizumab treatment among patients with higher PD-L1 expression levels was observed in a previous study; PFS was longer in patients with a TPS≥50% than those with a TPS 1–49% or a TPS<1%, while PFS was comparable between the three cohorts within the first 2 months [7]. These data suggested that PD-L1 expression levels could predict an immunotherapy benefit in the late phase, however it could not classify the subgroup which had treatment failure in the early phase.

The response rate to first-line pembrolizumab therapy was 50.3% in the present study, which was similar to the response rate observed among a PD-L1 TPS≥50% population in previous studies (44.8% to 50.0%) [7,8]. The present study revealed that a TPS 90–100% cohort had a numerically higher response rate than a TPS 50–89% cohort (58.0% vs 46.5%), which was close to the response rate (60.3% to 61.4%) among patients with a TPS≥50% treated with pembrolizumab plus chemotherapy [13,14]. In a previous study [7], the response rate for patients with a TPS≥50% exceeded the patients with both TPS 1–49% and TPS<1% for previously treated patients (ORR = 43.9%, ORR = 15.6%, ORR = 9.1%, *p*<0.001) and untreated patients (ORR = 50.0%, ORR = 19.2%, ORR = 16.7%, *p* = 0.01), while there was not a large difference between the patients with a TPS 1–49% and those with a TPS<1%. These data suggest that PD-L1 expression levels are associated with the response rate, but that they could not act as a biomarker to clearly classify NSCLC patients into responder or non-responder groups for pembrolizumab. Additionally, this study revealed that the TPS 90–100% cohort experienced a longer TTF than the TPS 50–89% cohort among the patients who had a response to pembrolizumab, whereas it was not evident among those who did not. Therefore, it is important to develop the tools to identify the subgroup that responds to pembrolizumab prior to administration of treatment.

A blockade of the PD-1/PD-L1 pathway restores effector T-cell function and enhances anti-tumor immune responses [15], suggesting that tumor PD-L1 expression is only the surrogate maker of effector T-cell activity. PD-L1 is an inducible molecule and PD-L1 expression levels are heterogeneous within tumors [16,17]. Moreover, the PD-1/PD-L1 pathway also plays a role in the expansion and functionality of regulatory T cells [18], and PD-L1 expression alone does not accurately assess the dynamic immune microenvironment. However, PD-L1 expression levels are the only predictive biomarkers of the clinical benefits from ICIs in the real world. Additional diagnostic approaches, including assessment of the genomic landscape and the presence of preexisting CD8+ T cells and cytokines in tumor samples, could supplement PD-L1 expression as a means of identifying patients who might have a response to ICIs [19]. One emerging biomarker of response to immunotherapy is the tumor mutational burden (TMB) which is associated with the clinical benefits of ICIs [10,20,21]. TMB is independent of PD-L1 expression with a similar predictive capacity, and a composite of both variables might be helpful in identifying with precision patients most likely to benefit [10]. Further studies are needed to develop the tools to identify such a subgroup.

A few limitations of the present study warrant mentioning. First, information bias was inevitable, given the retrospective nature of the study. Thus, caution is needed when interpreting the data and making generalizations to other cohorts. In addition, scheduled computed tomography or magnetic resonance imaging was not performed in this study, raising the possibility that we could not evaluate PFS precisely. Therefore, we analyzed clinical benefits of the treatment using TTF rather than PFS in this study. Second, the follow-up period was not sufficient to investigate long-term data. Therefore, we do not show the data on overall survival. Third, biomarkers other than PD-L1 (e.g., TMB) were not analyzed. However, these biomarkers are currently under active investigation.

## Conclusions

The patients with a TPS 90–100% continued first-line pembrolizumab for longer than those with a TPS 50–89%; this was driven by a reduced risk of treatment failure in the late phase, suggesting that PD-L1 expression levels might be a predictive biomarker of immunotherapy benefit in the late phase among NSCLC patients with a TPS ≥50%. However, confirmation in other cohorts is required, and there is a need to develop clinically practical tools to identify the subgroups of patients most likely to derive clinical benefits from immunotherapy in this population.

## Supporting information

S1 Fig Progression-free survival for pembrolizumab: The tumor proportion score 90–100% cohort versus the tumor proportion score 50–89% cohort. Panel A shows Kaplan–Meier survival curves for progression-free survival according to the programed death ligand-1 expression levels in the tumor proportion score 90–100% cohort versus the tumor proportion score 50–89% cohort. Panel B shows the Kaplan–Meier survival curves for progression-free survival before and after 120 days according to the programed death ligand-1 expression level in the landmark analyses. Hazard ratios are for the tumor proportion score 90–100% cohort versus the tumor proportion score 50–89% cohort. The hazard ratios, 95% confidence intervals, and p-values were calculated using univariate Cox regression analysis. Cross marks represent data censored at the last time the patient was known to be alive. Abbreviations: HR, hazard ratio; TPS, tumor proportion score.
(TIF)

S2 Fig Time to pembrolizumab treatment failure: The tumor proportion score 90–100% cohort versus the tumor proportion score 50–89% cohort among patients who had a response to pembrolizumab and those who did not. Kaplan–Meier survival curves for time to treatment failure according to the programed death ligand-1 expression levels (the tumor proportion score 90–100% cohort versus the tumor proportion score 50–89% cohort) and the response to pembrolizumab monotherapy. Hazard ratios are for the tumor proportion score 90–100% cohort versus the tumor proportion score 50–89% cohort among patients who had a response to pembrolizumab and those who did not. The hazard ratios, 95% confidence intervals, and p-values were calculated using univariate Cox regression analysis. Cross marks represent data censored at the last time the patient was known to be alive. Abbreviations: HR, hazard ratio; TPS, tumor proportion score.
(TIFF)

S3 Fig Time to treatment failure of pembrolizumab: The tumor proportion score 90–100% cohort versus the tumor proportion score 50–89% cohort in landmark analysis. Panel A and B shows the Kaplan–Meier survival curves for time to treatment failure before and after 60 days and 180 days according to the programed death ligand-1 expression levels in landmark analyses. Hazard ratios are for the tumor proportion score 90–100% cohort versus the tumor proportion score 50–89% cohort. The hazard ratios, 95% confidence intervals, and p-values were calculated using univariate Cox regression analysis. Cross marks represent data censored at the last time the patient was known to be alive. Abbreviations: HR, hazard ratio; TPS, tumor proportion score.
(TIF)
