# Supplementary figures and images for: Clinical outcomes in non-small cell lung cancer patients with an ultra-high expression of programmed death ligand-1 treated using pembrolizumab as a first-line therapy: A retrospective multicenter cohort study in Japan
Source: PLoS One. 2019 Jul 31;14(7):e0220570. doi: 10.1371/journal.pone.0220570 (PMC6668842; doi:10.1371/journal.pone.0220570)

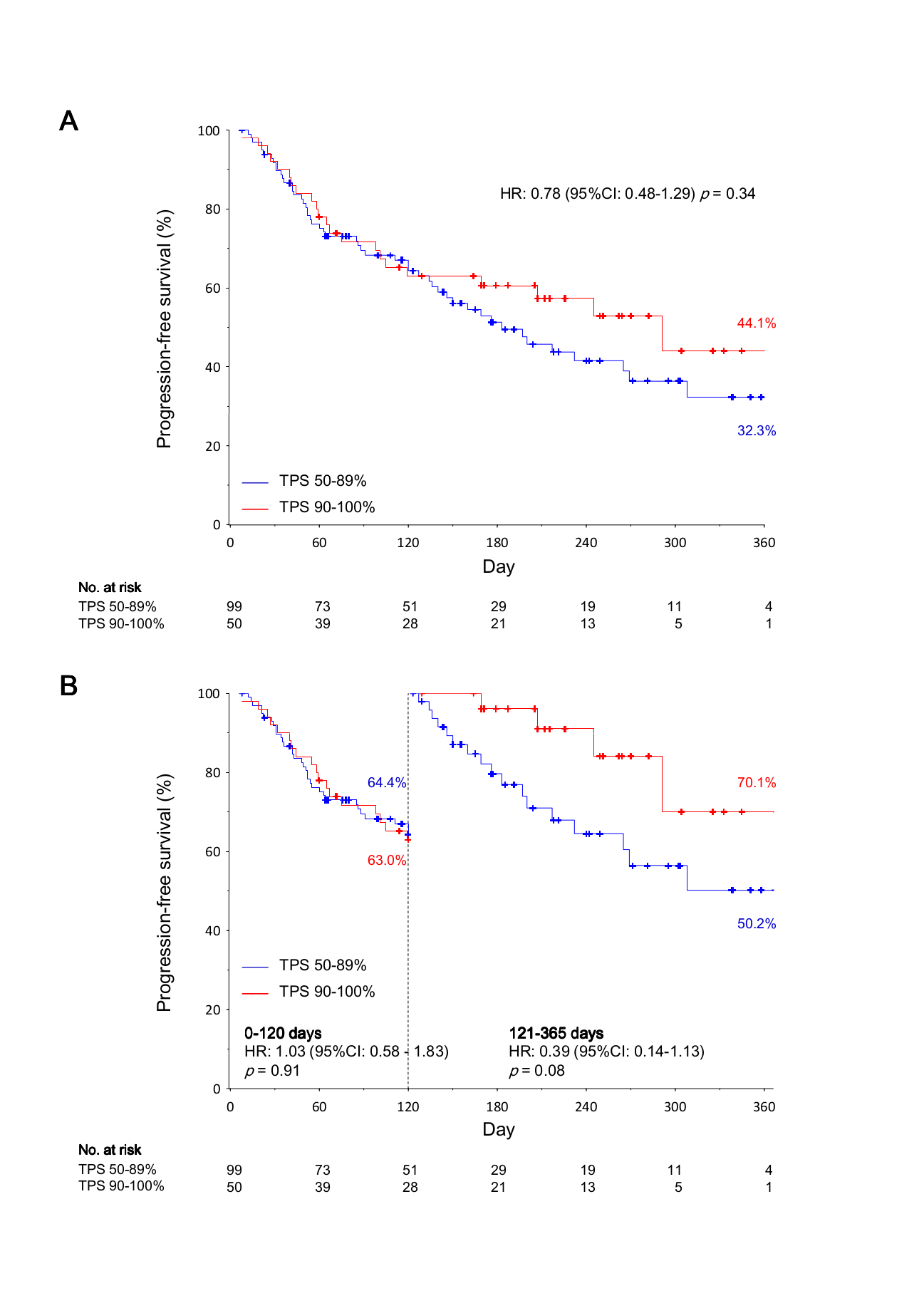

Supplement: S1 Fig — Panel A shows Kaplan–Meier survival curves for progression-free survival according to the programed death ligand-1 expression levels in the tumor proportion score 90–100% cohort versus the tumor proportion score 50–89% cohort. Panel B shows the Kaplan–Meier survival curves for progression-free survival before and after 120 days according to the programed death ligand-1 expression level in the landmark analyses. Hazard ratios are for the tumor proportion score 90–100% cohort versus the tumor proportion score 50–89% cohort. The hazard ratios, 95% confidence intervals, and p-values were calculated using univariate Cox regression analysis. Cross marks represent data censored at the last time the patient was known to be alive. Abbreviations: HR, hazard ratio; TPS, tumor proportion score. (TIF) [file pone.0220570.s001.tif]

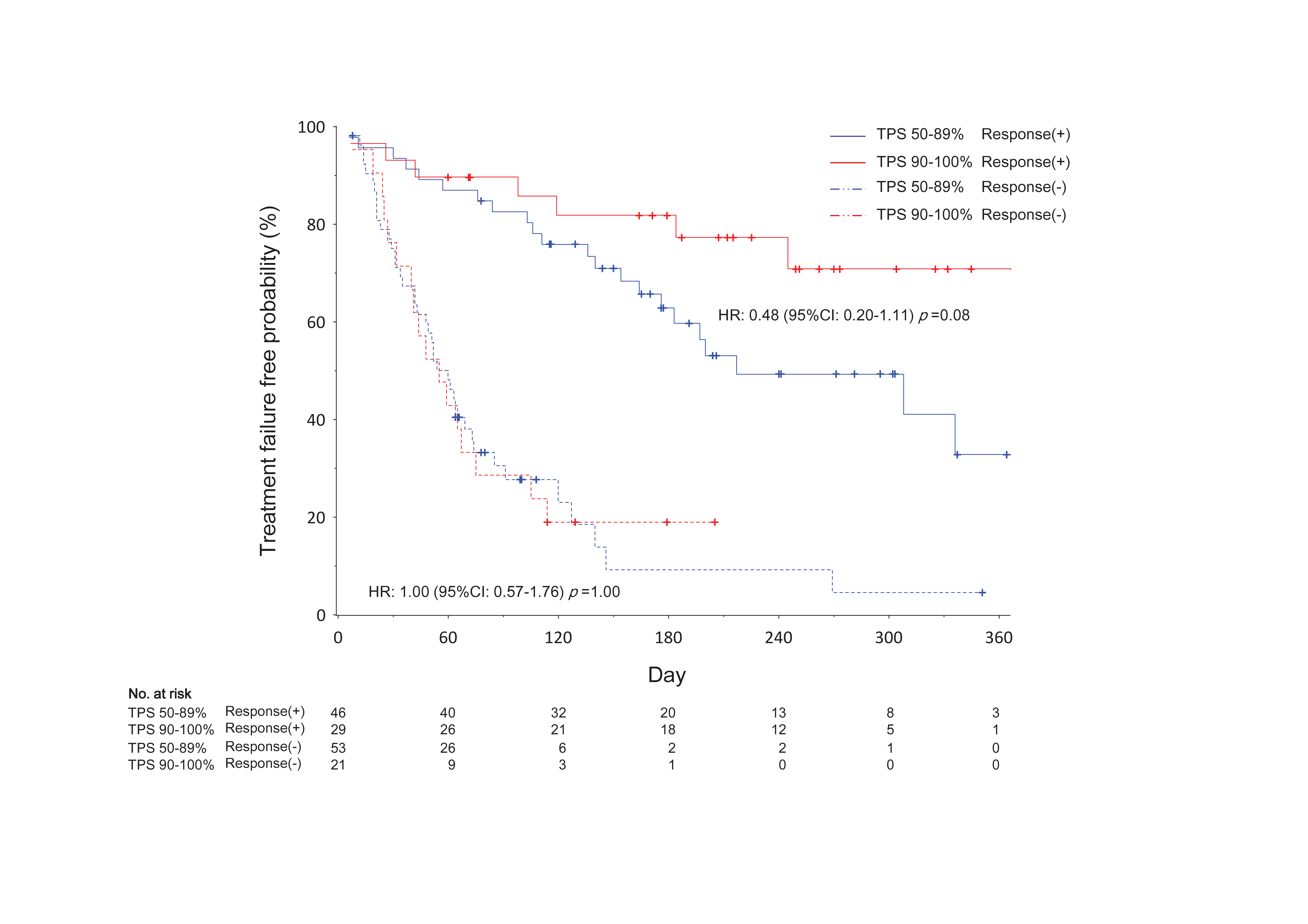

Supplement: S2 Fig — Kaplan–Meier survival curves for time to treatment failure according to the programed death ligand-1 expression levels (the tumor proportion score 90–100% cohort versus the tumor proportion score 50–89% cohort) and the response to pembrolizumab monotherapy. Hazard ratios are for the tumor proportion score 90–100% cohort versus the tumor proportion score 50–89% cohort among patients who had a response to pembrolizumab and those who did not. The hazard ratios, 95% confidence intervals, and p-values were calculated using univariate Cox regression analysis. Cross marks represent data censored at the last time the patient was known to be alive. Abbreviations: HR, hazard ratio; TPS, tumor proportion score. (TIFF) [file pone.0220570.s002.tiff]

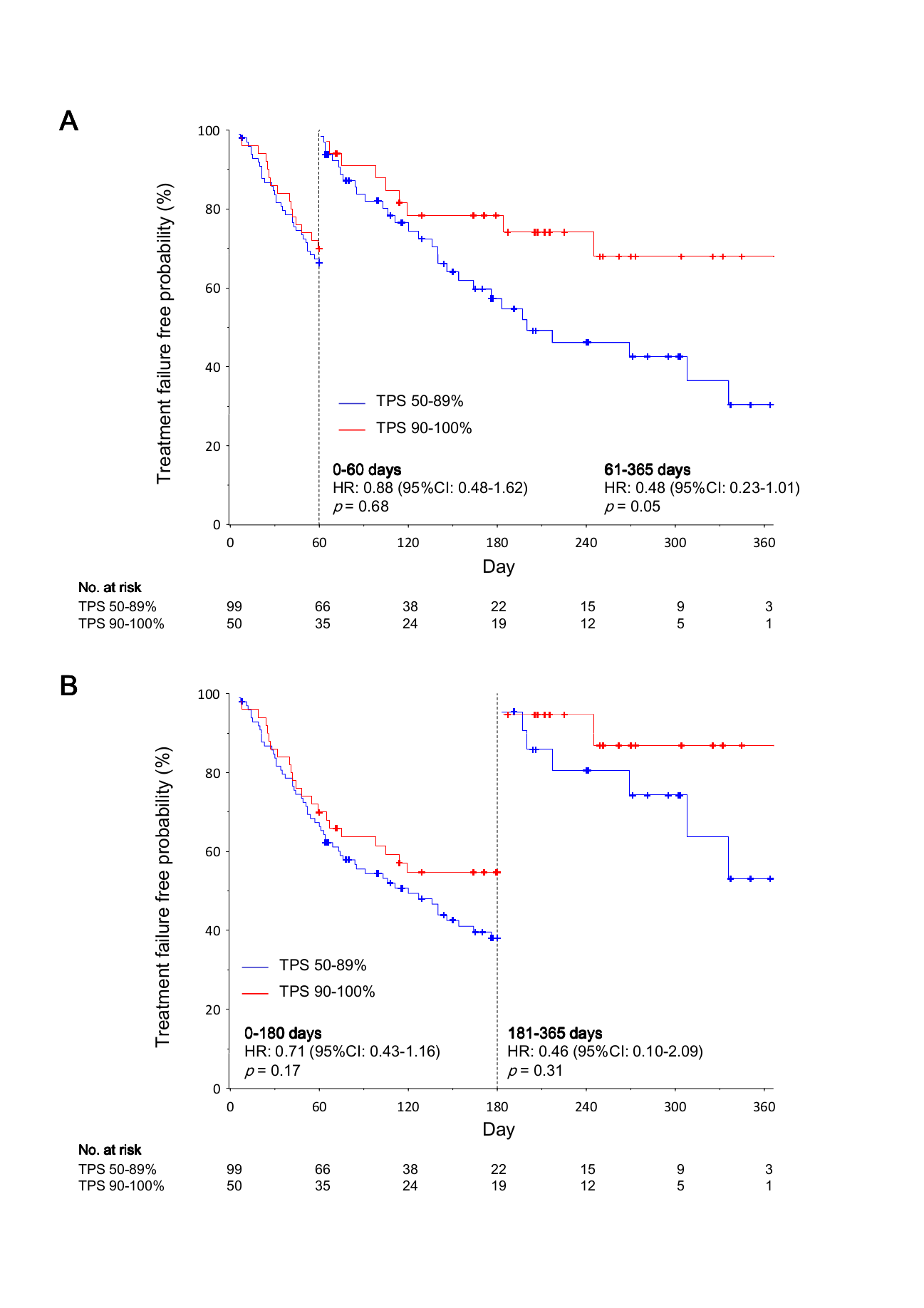

Supplement: S3 Fig — Panel A and B shows the Kaplan–Meier survival curves for time to treatment failure before and after 60 days and 180 days according to the programed death ligand-1 expression levels in landmark analyses. Hazard ratios are for the tumor proportion score 90–100% cohort versus the tumor proportion score 50–89% cohort. The hazard ratios, 95% confidence intervals, and p-values were calculated using univariate Cox regression analysis. Cross marks represent data censored at the last time the patient was known to be alive. Abbreviations: HR, hazard ratio; TPS, tumor proportion score. (TIF) [file pone.0220570.s003.tif]
